# THE “EPIDEMIC” OF OLDER ADULT LONELINESS: PROBLEMS OF DIAGNOSTIC INTERVENTION AND CRITICAL SOCIOLOGICAL RESEARCH

**DOI:** 10.1093/geroni/igae098.1970

**Published:** 2024-12-31

**Authors:** Stephen Katz

**Affiliations:** Trent University, Toronto, Ontario, Canada

## Abstract

Loneliness, more than a feeling, has become a new ‘geriatric giant’ (Freedman & Nicolle 2020) and epidemic health crisis for older adults, affecting their physical, cognitive and emotional well-being. During the COVID epidemic, the intersecting effects of loneliness and isolation (often blurred in the literature) have intensified, as varying public health measures restricted visiting, gathering, routines and activities. While technical interventions, such as digital communication technologies (DCTs), tele-health meetings, online games, robotic pet companions and simulated presence therapy (SPT) are offered as beneficial aids, even where available or co-designed they tend to individualize and universalize loneliness. Professional, recreational and prescriptive interventions can also disregard the structural relations and socio-material environments that configure everyday lonely-making experiences over time. For both residential and community dwelling older adults, such experiences include lack of affordable housing, care-giver burden and insufficient community resources and planning. This presentation, drawing upon data and examples from senior health policy, loneliness surveys, national reports and qualitative research, reflects on these troubling matters in their complexity and heterogeneity. Conclusions explore the making of an ageist emotional economy that depicts and neglects older adults as inevitably lonely, while advocating for their rights to age in safe, healthy and socially connective ways.

